# Chronic hepatitis C liver microenvironment: role of the Th17/Treg interplay related to fibrogenesis

**DOI:** 10.1038/s41598-017-13777-3

**Published:** 2017-10-16

**Authors:** Daniela Alejandra Rios, Pamela Valva, Paola Cecilia Casciato, Silvia Frias, María Soledad Caldirola, María Isabel Gaillard, Liliana Bezrodnik, Juan Bandi, Omar Galdame, Beatriz Ameigeiras, Diana Krasniansky, Carlos Brodersen, Eduardo Mullen, Elena Noemí De Matteo, María Victoria Preciado

**Affiliations:** 1 Instituto Multidisciplinario de Investigaciones en Patologías Pediátricas (IMIPP- CONICET-GCBA) Laboratorio de Biología Molecular, División Patología, Hospital de Niños Ricardo Gutiérrez, Gallo 1330, C1425EFD Buenos Aires, Argentina; 2 Unidad de Hepatología, Hospital Italiano de Buenos Aires; Juan D Perón 4190, C1181ACH Buenos Aires, Argentina; 3 Unidad de Hepatología, Hospital Ramos Mejía; Urquiza 609, CP1221 Buenos Aires, Argentina; 4 Instituto Multidisciplinario de Investigaciones en Patologías Pediátricas (IMIPP- CONICET-GCBA), Departamento de Inmunología, Hospital de Niños Ricardo Gutiérrez; Gallo, 1330 C1425EFD Buenos Aires, Argentina; 5 Unidad de Hepatología, Hospital General de Agudos “Carlos G. Durand”; Av Díaz Vélez 5044, C1405DCS Buenos Aires, Argentina; 6 División Patología, Hospital Italiano de Buenos Aires; Juan D Perón 4190, C1181ACH Buenos Aires, Argentina

## Abstract

The role of the different lymphocyte populations in liver microenvironment of chronic hepatitis C (CHC) patients is still matter of debate. Since Th17 and Treg have opposite functions, their balance could affect disease progression. The aim was to explore liver microenvironment and its peripheral blood counterpart in adult CHC patients. CD4^+^ lymphocytes were predominant in the liver, with high Foxp3^+^ but low IL-17A^+^ frequency. IL-17A^+^ lymphocytes and IL-17A^+^/Foxp3^+^ ratio displayed association with advanced fibrosis (*p* = 0.0130; *p* = 0.0236, respectively), while Foxp3^+^ lymphocytes and IL-10 expression level inversely correlated with fibrosis severity (*p* = 0.0381, *p* = 0.0398, respectively). TGF-β/IL-6 ratio correlated with IL-17A^+^/Foxp3^+^ ratio (*p* = 0.0036, *r* = 0.5944) and with IL-17A^+^ lymphocytes (*p* = 0.0093; *r* = 0.5203). TNF-α and TGF-β were associated with hepatitis severity (*p* = 0.0409, *p* = 0.0321). Peripheral blood lymphocyte frequency was not associated with liver damage. There are functionally different immune cell populations actively involved in liver damage, but the liver cytokine milieu actually drives the pathogenesis. The intrahepatic Foxp3^+^ lymphocytes predominance beside the low IL-17A^+^ lymphocytes frequency, delineate a skewed IL-17A^+^/Foxp3^+^ balance towards Foxp3^+^ lymphocytes. However, the IL-17A^+^ lymphocytes association with advanced fibrosis denotes their role in the pathogenesis. Therefore, the interplay between Th17 and Treg conditions liver fibrogenesis.

## Introduction

Hepatitis related to Hepatitis C virus (HCV) is a progressive disease, so liver failure as a consequence of HCV infection is one of the most common reasons for liver transplantation. It is considered that most of the infected patients are unable to clear the virus and will develop chronic infection in the face of the on-going innate and adaptive immune response^1^.

Chronic hepatitis C (CHC) still represents a major global health problem since over 115 million persons are infected worldwide and there is no available vaccine^2^. Although the recently approved direct-acting antiviral agents (DAA) have a dramatically higher sustained virologic response (SVR) rate, they are still poorly accessible due to their high costs and the real burden of HCV infection displays great uncertainty^2^. Currently, one of the major defiance is to carry out screening programs to assess HCV prevalence in the context of an asymptomatic infection^3^. Another critical point is that the “virological cure” does not necessarily mean that the patients are cured of liver disease or protected against the development of potential HCC, consequently the mechanisms leading to liver injury in CHC are under constant revision^4^. The fact that both immune system-mediated reactions and viral cytopathic effect are involved in pathogenesis is widely accepted^5,6^, thus, the interplay between virus and host immune response may influence infection outcome.

Blood CD4^+^ and CD8^+^ T cells response have limited impact on disease course whereas intrahepatic T cells seem to control disease pathogenesis^7,8^. Therefore, their regulation in HCV patients may have important implications not only in determining spontaneous clearance but also in disease progression. Normally, cytotoxic T lymphocytes (CTL, CD8^+^ T cells) are essential for infection control, as they migrate to infected tissues and mediate viral clearance. However, HCV-specific CTLs in CHC patients has a lesser capacity to proliferate and produce IFN-γ in response to viral antigens^9^. T helper lymphocytes (Th, CD4^+^ T cells) act as central regulators of the adaptive immune response through augmenting CTL response and antigen-specific B lymphocytes (BL). It is well known that Th can be induced to differentiate towards Th1 (Tbet^+^/IFN-γ), Th2 (GATA3^+^/IL-4), Th17 (ROR-γ^+^/IL-17) and Treg (CD25^hi^ CD127^low^ Foxp3^+^/IL-10 TGF-β) cells with distinct phenotypes and functions. Th1 participates in cell-mediated immune response by secreting IFN-γ, TNF-α, and IL-2. Th17 lymphocytes represent a pro-inflammatory subset, which contribute to autoimmunity and tissue damage, while Tregs control the balance between immune activation and tolerance, by cell to cell contact or secreting IL-10 and TGF-β^10^. Nevertheless, further studies are necessary to clarify in which way the immune system is participating in the pathogenesis of CHC and which is the role of each T lymphocytes subset. Hence, to understand HCV pathogenesis, further investigations in patient’s samples instead of animal models are needed, since it is crucial to explore different infection aspects into its actual context. Moreover, HCV immune response has been mainly studied in peripheral blood mononuclear cells (PBMC) samples because of the difficulty in obtaining HCV-infected liver tissue.

We have previously demonstrated, in a comparative study between a pediatric and adult CHC cohorts that HCV, apoptosis and immune response are all involved in CHC pathogenesis; however, there are certain differences between children and adults regarding the role played by each component on the final scenario^6^. Liver injury in pediatric CHC would be largely associated with viral cytopathic effect mediated by apoptosis, while in adults it would be mainly associated with an exacerbated immune response. Taking this into account; we decided to explore the immune pathogenesis in a new cohort of CHC adult patients^6^. Therefore, in this study, the inflammatory intrahepatic microenvironment, comprising both cells and cytokines, was assessed in order to get a more comprehensive picture of the relation between immune response and CHC damage. In addition, since several extrahepatic manifestations associated to CHC infection have been reported^11^, it was also explored to what extent the peripheral blood (PB) compartment mirrors the hepatic immune response.

## Results

### Characterization of liver microenvironment

**Figure 1:**
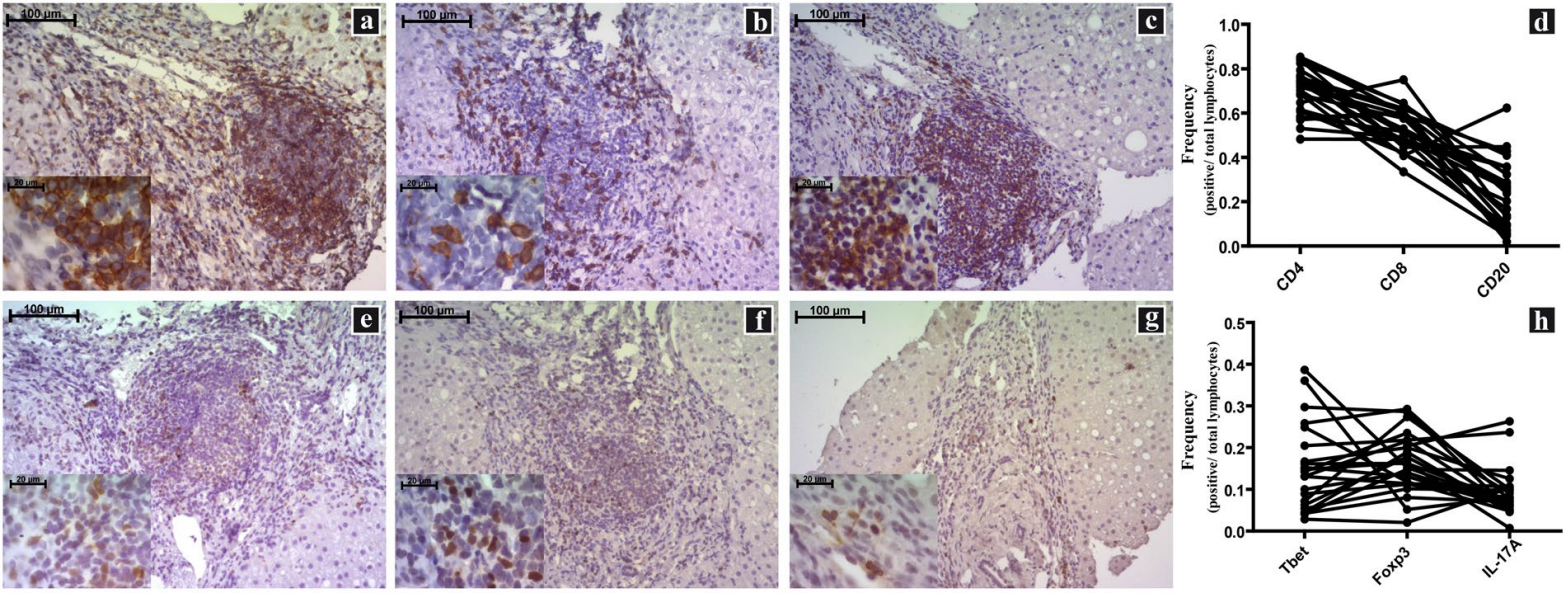
Immunostaining of P-P/I lymphocyte populations on formalin fixed liver biopsies. (**a**) CD4^+^ lymphocytes, (**b**) CD8^+^ lymphocytes, (**c**) CD20^+^ lymphocytes, (**d**) CD4^+^, CD8^+^ and CD20^+^ lymphocytes frequency for each patient, (**e**) Tbet^+^ lymphocytes, (**f**) Foxp3^+^ lymphocytes, (**g**) IL-17A^+^ lymphocytes, (**h**) Tbet^+^, Foxp3^+^ and IL-17A^+^ lymphocytes frequency for each patient. Frequencies were calculated as immunostained P-P/I lymphocytes/ total P-P/I lymphocytes in all portal tracts of the tissue section (400×).

**Figure 2:**
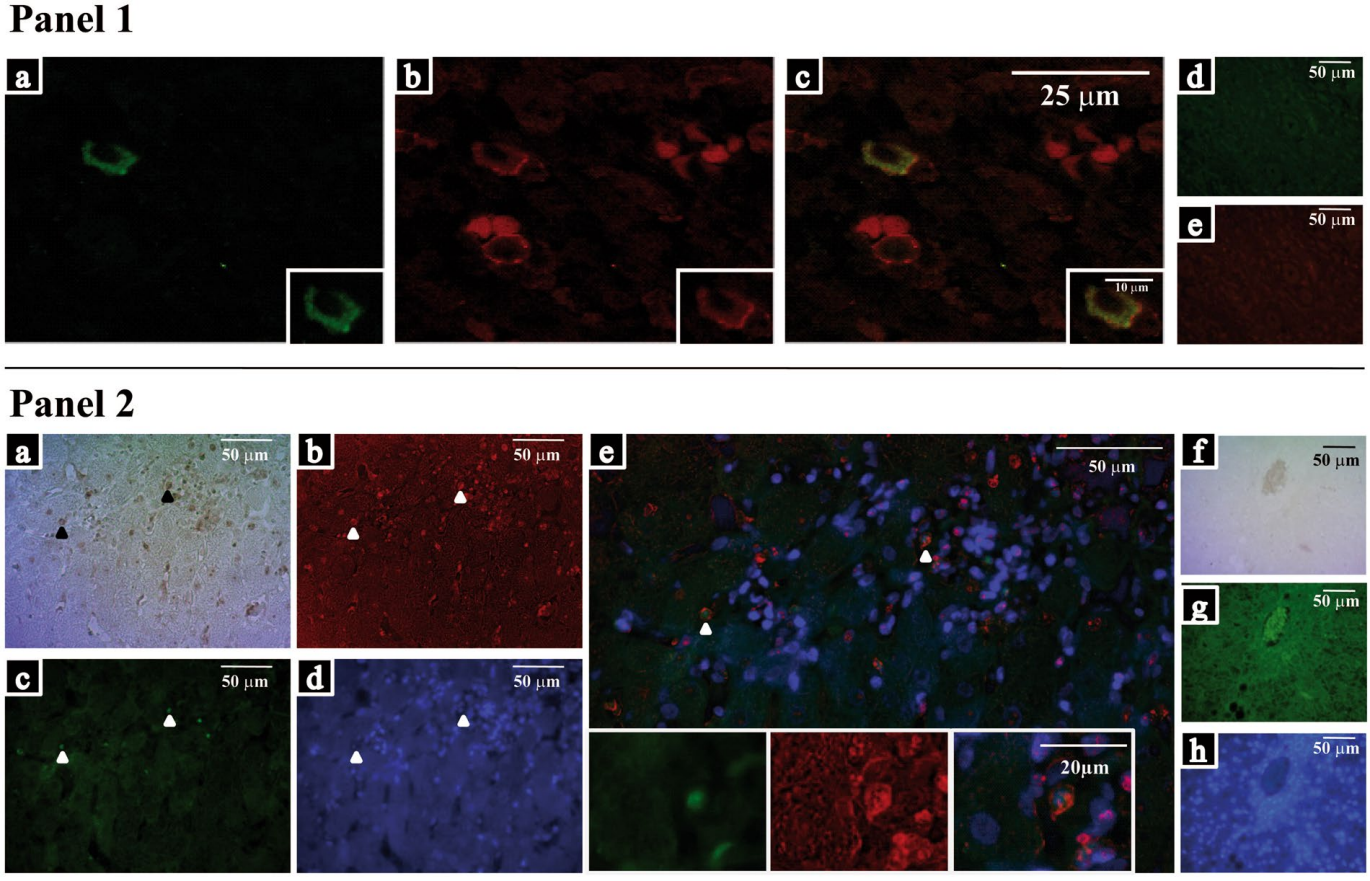
Double CD4/IL-17A and CD4/Foxp3 immunostainings. Panel 1: Confocal microscopy image of CD4^+^/IL-17A^+^ lymphocytes. (**a**) IL-17A^+^ lymphocytes, (**b**) CD4^+^ lymphocytes, (**c**) merged images (CD4 show a membrane immunostaining while IL-17A show a cytoplasmic one). The insertions show image amplification. (**d**) and (**e**) IL-17A and CD4 isotype control images, respectively. Panel 2: Epifluorescence light microscopy image CD4^+^/Foxp3^+^ lymphocytes. (**a**) CD4^+^ lymphocytes, (**b**) false red colour, (**c**) Foxp3^+^ lymphocytes, (**d**) DAPI counterstaining and (**e**) merged images (the insertion shows image amplification). (**f**), (**g**) and (**h**) isotype control images.

The liver microenvironment was analysed on formalin-fixed and paraffin-embedded (FFPE) as well as fresh biopsies samples from CHC patients using two complementary approaches. Clinical, virological and histological patients’ features are described in Table 1. The first approach comprises the assessment of CD4^+^, CD8^+^ and CD20^+^ lymphocytes as well as CD4^+^ subsets on liver biopsies (Fig. 1 and Supplementary Fig. 1). All the studied populations were predominantly identified in the portal and periportal/interface infiltrate (P-P/I) areas while only scattered lymphocytes were observed in the intralobular region. Lymphocyte distribution turned out to be an interesting finding, since CD4^+^ lymphocytes were present in the centre and interface areas of the portal infiltrates, while CD8^+^ lymphocytes showed a peripheral localization within hepatic lymphoid aggregates (Supplementary Fig. 2). Concerning the frequencies of the different populations, CD4^+^ lymphocytes were predominant [median: 0.717 (range: 0.482–0.852)] followed by CD8^+^ [0.520 (0.335–0.750)] and CD20^+^ lymphocytes [0.238 (0.019–0.623)] (Fig. 1d). When analysing the frequencies in CD4^+^ subsets, IL-17A^+^ [0.075 (0.007–0.263)] was the lowest {Foxp3^+^ [0.162 (0.021–0.293)] and Tbet^+^ [0.137 (0.029–0.386)]}. It’s remarkable that the analysis on a case-by-case basis displayed a clear Foxp3^+^ predominance over IL-17A^+^ lymphocytes (Fig. 1h). Since there are other lymphocyte populations producing IL-17A and Foxp3, IL-17A^+^/CD4^+^ as well as Foxp3^+^/CD4^+^ lymphocytes were identified by double immunostaining (Fig. 2).

**Table 1:** Clinical, virological and histological patient features.

		Chronic HCV Patients (n = 27)
Age (ys) median (range)		53 (32–72)
Sex	Male % (n)	51.9 (14)
Risk factor for HCV infection % (n)	Drug abuse	14.8 (4)
	Transfusion	22.2 (6)
	Sexual	11.1 (3)
	Haemodialysis	3.7 (1)
	Occupational	3.7 (1)
	Tattoos	3.7 (1)
	Unknown	3.7 (11)
Genotype % (n)	1a	33.3(9)
	1b	44.4 (12)
	2	3.7 (1)
	3a	7.4 (2)
	ND	11.1 (3)
Viral load (IU/ml) median		888,240
(range)		(12,535–9,070,000)
ALT (IU/L) median (range)		82 (23–254)
% (n)	Elevated	92.6 (25)
AST (IU/L) median (range)		64.5 (22–234)
% (n)	Elevated	81.5 (22)
Hepatitis % (n)	Minimal	3.70 (1)
	Mild	18.52 (5)
	Moderate	62.96 (17)
	Severe	14.82 (4)
Fibrosis stage^1^% (n)	Significant Fibrosis (≥2)	62.96 (17)
	Advanced Fibrosis (≥3)	37.04 (10)

ND: not determined. ALT: alanine aminotransferase. AST: aspartate aminotransferase (Normal ALT levels in adult patients were ≤ 32 and normal AST ≤ 48 UI/L when test was done at 37 °C). ^1^Fibrosis according to METAVIR.

In the second approach, pro-inflammatory cytokines IL-17A, IL-6, IFN-γ and TNF-α, as well as anti-inflammatory IL-10 and TGF-β were quantified to evaluate liver milieu secretion activity. Each cytokine displayed its own expression level in the liver. On this regard, IL-17A had the lowest level which agreed with the lowest frequency of IL-17A^+^ lymphocytes showed by immunohistochemistry. None of the evaluated cytokines showed a correlation with any specific cytokine producer lymphocyte population of the infiltrate.

It has been demonstrated that the imbalance between Treg and Th17 cells might play an important role in the course of inflammatory diseases as well as in persistent HCV infection, since Tregs show an immunosuppressive phenotype while Th17 are pro-inflammatory. On the other hand, they present a kind of plasticity in the differentiation process, according to the cytokine microenvironment particularly depending on TGF-β and IL-6^10,12^. In line with this, we evaluated IL-17A^+^/Foxp3^+^ lymphocytes ratio in relation to the above mentioned cytokines. Positive correlations of TGF-β/IL-6 with IL-17A^+^/Foxp3^+^ lymphocytes ratio (*p* = 0.0036, *r* = 0.5944) as well as with IL-17A^+^ lymphocyte frequency (*p* = 0.0093; *r* = 0.5203) were observed (Supplementary Fig. 3). Oddly enough, no correlation between TGF-β and either Foxp3^+^ or IL-17A^+^ lymphocyte frequency were found. The IL-6 mRNA level did not correlate with IL-17A^+^ lymphocyte frequency either. TNF-α, IFN-γ and TGF-β showed a positive correlation each other, so they seemed to be tightly related (Supplementary Fig. 3). Finally, it was interesting to note that IL-17A^+^ lymphocyte frequency as well as IFN-γ and TGF-β levels positively correlated with viral load (*p* = 0.030, *r* = 0.427; *p* = 0.024, *r* = 0.472; *p* = 0.011, *r* = 0.511; respectively) denoting the interrelation among liver-virus-immune system (Supplementary Fig. 3).

### Liver microenvironment in relation to liver damage

**Figure 3:**
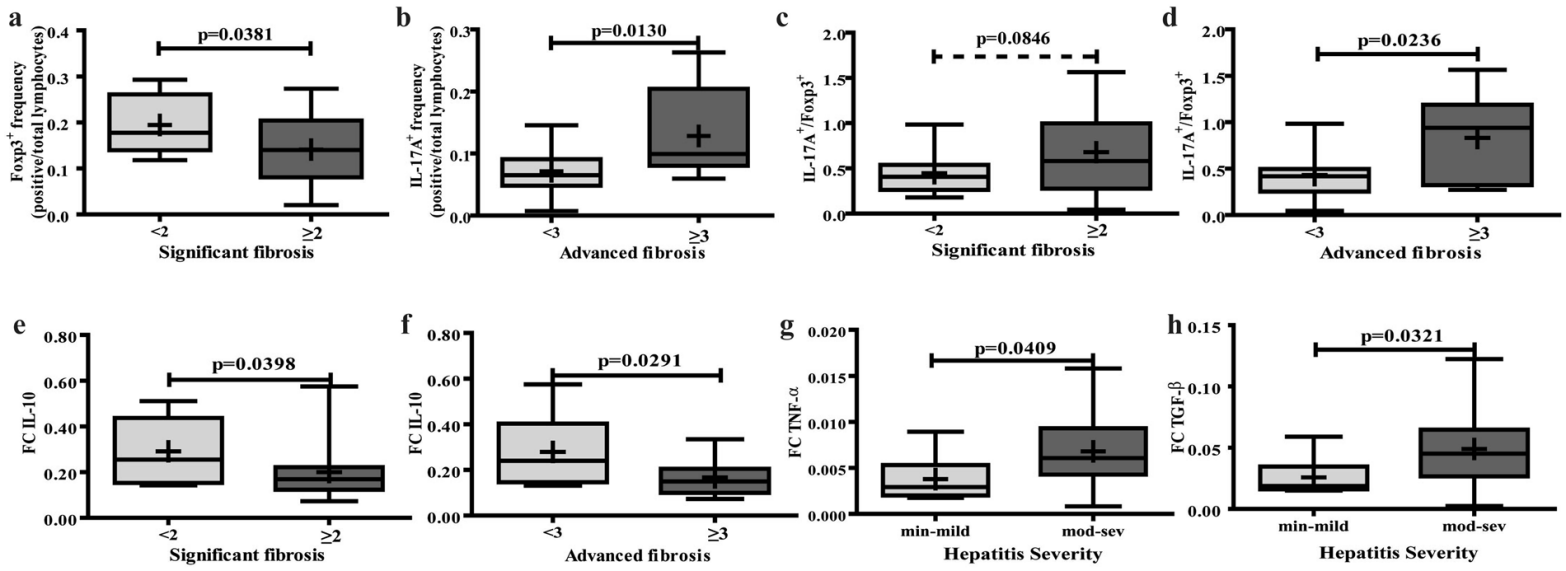
Liver microenvironment related to liver damage. Association between: (**a**) Foxp3^+^ lymphocyte frequency and significant fibrosis, (**b**) IL-17A^+^ lymphocyte frequency and advanced fibrosis, (**c**) IL-17A^+^/Foxp3^+^ lymphocytes ratio and significant fibrosis (trend of association), (**d**) IL-17A^+^/Foxp3^+^ lymphocytes ratio and advance fibrosis, (**e**–**f**) IL-10 expression level with significant and advanced fibrosis, respectively, (**g**–**h**) TNF-α and TGF-β expression level with hepatitis severity, respectively. Significant (F ≥ 2) and advanced (F ≥ 3) fibrosis according to METAVIR. Hepatitis severity (min: minimal; mod: moderate, sev: severe) according to HAI. The results are depicted in box plots. Horizontal lines within boxes indicate medians. Horizontal lines outside the boxes represent the 5 and 95 percentiles. Mean is indicated as + . Frequencies were calculated as immunostained P-P/I lymphocytes/ total P-P/I lymphocytes in all portal tracts of the tissue section (400×). FC: fold change. The unpaired t-test (**a**,**c**,**f**,**g** and **h**) and Mann-Whitney U-test (**b**,**d** and **e**) were used to compare sets of data.

To understand the role of immune response in CHC, we evaluated the frequency of intrahepatic cell populations related to biochemical and histological parameters. Portal Foxp3^+^ and IL-17A^+^ lymphocyte frequency as well as the IL-17A^+^/Foxp3^+^ lymphocytes ratio displayed association with fibrosis severity. While Foxp3^+^ lymphocytes frequency was higher in biopsies with lower stage of fibrosis (*p* = 0.0381), IL-17A^+^ lymphocytes and the IL-17A^+^/Foxp3^+^ lymphocytes ratio showed association with advanced fibrosis (*p* = 0.0130; *p* = 0.0236, respectively) (Fig. 3 and Supplementary Fig. 4). Of note was the fact that IL-17A^+^ and Foxp3^+^ lymphocytes were also present in the intralobular component, but their frequency did not show association with liver damage parameters, which reflects that their interplay at the portal tract locally regulates fibrosis progression. Concerning the other P-P/I lymphocyte populations, CD8^+^, CD20^+^, CD4^+^ and Tbet^+^, no association with any histological parameter was observed.

When considering biochemical parameters of liver damage, IL-17A^+^ lymphocytes frequency, IL-17A^+^/Foxp3^+^ lymphocytes ratio as well as CD8^+^ lymphocytes positively correlated with transaminases levels (*p* = 0.0003*, r* = 0.6682*; p* = 0.0021*, r* = 0.5976; *p* = 0.0147*, r* = 0.4647, respectively) (Supplementary Fig. 3).

With reference to liver cytokine milieu, IL-10 levels showed an inverse relation with fibrosis severity (significant fibrosis *p* = 0.0398, advanced fibrosis *p* = 0.0291) whereas TNF-α and TGF-β were associated with hepatitis severity (*p* = 0.0409, *p* = 0.0321; respectively) (Fig. 3). The other assessed cytokines displayed no association with liver damage parameters (Supplementary Fig. 5).

### Peripheral compartment

All the immune cell populations tested in the liver were also determined by flow cytometry in PB of CHC patients. In addition, samples from healthy donors were also included in this analysis, but no differences in lymphocyte populations’ frequency were depicted between patients and donors (Table 2); suggesting a liver localized inflammatory process. Moreover, none of the peripheral immune cell population detected was associated with liver damage, transaminases or viral load.

**Table 2:** Evaluation of peripheral lymphocyte population in CHC patients vs uninfected controls.

	Cell frequency		*P* value
	Donor	HCV^+^	
Th	46.0 (29.0–51.0)	44.5 (18.0–68.0)	0.339
CTL	25.00 (19.0–36.0)	26.0 (11.0–63.0)	0.376
BL	10.0 (7.0–13.0)	12.0 (3.0–25.0)	0.103
Th1	3.59 (0.3–10.5)	3.6 (1.8–6.6)	0.491
Treg	4.7 (2.1–8.3)	5.1 (2.5–9.9)	0.408
Th17	0.3 (0.1–1.6)	0.3 (0.1–1.0)	0.474

Results are expressed as median (range). Th: T helper lymphocytes, CTL: Cytotoxic T lymphocytes, BL: B lymphocytes.

The frequency profile of each peripheral and liver cell population in CHC was coincident; namely, Th > CTL > BL together with a higher level of Treg over Th17 was observed in both compartments (Supplementary Fig. 6). However, no correlation between compartments was observed for each population.

## Discussion

Healthy liver, as an immunological organ, is subject to a tightly regulated inflammation process which activates only when it needs to get rid of damaging agents^13^. However, in the context of CHC, it has been described a progressive functional exhaustion of certain immune cell populations^14^, which in an interplay with the virus could mediate liver damage. Nevertheless, the immune mechanisms underlying this failure are not well understood, so our aim was to characterize CHC immune pathogenesis of the liver. Of note, most studies dealing with this issue are mainly tested in PBMC samples, since liver biopsies are difficult to obtain, especially with the current non-invasive alternatives for liver fibrosis diagnosis^15^.

Th17 cells have recently been identified as a unique CD4^+^ T-helper subset characterized by IL-17A production and they are important contributors to hepatic inflammation and liver cirrhosis^16^. In contrast, Tregs have been identified as suppressors of diverse immune responses and inflammation. The alteration in Th17/Treg balance could affect disease progression since these two populations have opposite functions. In fact, this issue was recently reported in the context of persistent hepatitis B virus infection^17,18^. In our cohort, CHC biopsies displayed infiltrates with a large number of Foxp3^+^ cells as previously reported by Rushbrook *et al*.^19^ and Claassen *et al*.^20^, mainly located around portal tracts. Moreover, Claassen *et al*. showed a lower number of Foxp3^+^ lymphocytes in healthy donors than in HCV^+^ patients^20^, suggesting that Tregs are controlling immune response strength. Besides, in our series IL-17A^+^ lymphocytes were also found in a lower proportion in the liver denoting their contribution to the pathogenesis given its relation to liver damage parameters. Therefore, if there is a preferential homing from peripheral blood or a *de novo* generation process of these two cell populations in CHC pathogenesis, it is still a matter of debate.

Tregs and Th17 cells share mechanisms and key mediators at the differentiation process: TGF-β is essential for both Treg and Th17 lymphocyte differentiation in a concentration dependent manner whereas pro-inflammatory cytokines (IL-6, IL-1β or IL-21) mediate Th17 generation^12^. Then, an environment wherein both TGF-β and IL-6 are available promotes Th17 development^21^. In this cohort, positive correlations of TGF-β/IL-6 with IL-17A^+^/Foxp3^+^ lymphocytes ratio as well as with IL-17A^+^ lymphocyte frequency were described, despite there was no correlation between TGF-β expression and Foxp3^+^ lymphocyte frequency or between IL-6 expression and IL-17A^+^ lymphocyte frequency. Thus, based on the results from the current experiments, the cytokine liver milieu could contribute to the development or permanency of both cells types. The Foxp3^+^ lymphocytes predominance together with the low IL-17A^+^ lymphocyte frequency, delineate a skewed IL-17A^+^/Foxp3^+^ balance towards Foxp3^+^ lymphocytes. However, the association of IL-17A^+^ lymphocytes with advanced fibrosis might denote their role in the pathogenesis scenario.

Recent issues demonstrated that IL-17A production was not restricted to Th17 subset^22^, since various IL-17A producing immune cells were characterized, including CD8^+^ T cells, NK and NKT cells, macrophages, neutrophils, etc^23^. Malcek Jilkova *et al*. recently described an increasing proportion of IL-17A^+^ neutrophils out of total IL-17A^+^ cells, according to the fibrosis severity^23^. In our study, only IL-17A^+^ cells showing a clear lymphocyte morphology (easily recognized by pathologists) were quantified coupled with the demonstration of IL-17A^+^/CD4^+^ cells by double immunostaining.

In line with the regulatory microenvironment observed in these patients, both Foxp3^+^ lymphocyte frequency and IL-10 levels showed an association with lower fibrosis severity. This could indicate a higher Foxp3^+^ lymphocyte activity in early stages of disease progression. In accordance, Speletas *et al*. reported this correlation when patients with hepatitis of different aetiologies were evaluated^24^ and Aroucha *et al*. showed higher serum IL-10 in HCV^+^ patients with mild fibrosis compared to severe fibrosis^25^. In addition, it was demonstrated a decrease in liver fibrosis in CHC patients treated with recombinant IL-10^26^, which supports the protective role of IL-10 for developing fibrosis. The IL-10 may limit fibrogenesis by inhibiting collagen matrix deposition by hepatic stellate cells^27^ as well as by abolishing effector functions of other T cells thereby preventing hepatic stellate cells activation^28^. To sum up, this suggests that Foxp3^+^ lymphocytes and IL-10 secretion contribute to limit liver damage, mainly fibrosis, whereas the scant intrahepatic IL-17A^+^ lymphocytes are promoting liver fibrogenesis. However, Tregs role in CHC are still a matter of debate, since Sturm *et al*.^29^ reported positive correlation between intrahepatic CD4^+^-Foxp3^+^ frequency and fibrosis severity, and Langhans *et al*.^30^ described positive correlation of IL-8^+^-Foxp3^+^-CD4^+^ lymphocytes with worse fibrosis. Therefore, the liver regulatory milieu is probably a two-edged sword with a beneficial suppression of the unspecific immune response thereby limiting the harmful inflammation, but a detrimental suppression of the specific immune responses directed against HCV leading to the lack of virus clearance which in turn triggers disease progression^14^.

It would be worthwhile to mention that although Tbet^+^ lymphocytes were not associated with liver damage their frequency was similar to the Foxp3^+^ lymphocytes, so they might contribute to the cytokine liver milieu by producing IFN-γ.

Regarding pro-inflammatory cytokines present at the liver microenvironment, it is known that TNF-α production is induced and up-regulated in the liver by NS3^29^ and intervenes early in the cascade of events leading to liver damage. Meanwhile TGF-β is a pleiotropic cytokine that represents a link between immune response and fibrogenesis. In this series IFN-γ, TNF-α and TGF-β showed positive correlations among each other. In addition, TNF-α and TGF-β were associated with higher HAI values, which may represent their role in hepatitis regulation. In accordance, Sturm *et al*. described a positive correlation between TNF-α and TGF-β as well as between TGF-β and IL-10^29^. Interestingly, despite its known fibrogenic potential, TGF-β level did not show relation with fibrosis severity, which could be explained by its dual effect on fibrogenesis, activating stellate cells and regulating different immune cells functions^31^.

On the other side, most studies focus on peripheral immune cell population but the results are somewhat controversial, with some authors reporting elevated Treg^30,32^ and Th17^16,33^ while others describing low frequencies^20^. These contradictory results probably reflect differences in the design of immune assay, the sample tested (i.e. whole blood, PBMC) and the reference frame for reporting lymphocyte population frequency. In the studied group, CHC seemed like a localized inflammatory process since no difference in lymphocyte populations frequency was depicted between patients and donors as well as no correlation between liver damage parameters, transaminases levels or viral load were observed.

This study provides new insights in the role of the immune microenvironment in the complex process of CHC pathogenesis. There are several immune cell populations with different functions actively involved in liver damage, but the liver cytokine milieu could influence their effective participation in the pathogenesis. Our results interestingly highlight the role of the interplay between Th17 and Treg in the fibrogenesis process.

## Materials and Methods

### Patients and samples

Liver biopsies and concomitant PB samples were collected from 27 adult patients with chronic HCV infection naïve of treatment who received medical care at the Hospital Italiano de Buenos Aires, Hospital JM. Ramos Mejía and Hospital General de Agudos “Carlos G. Durand”. Liver samples were divided into two portions: one fragment was formalin-fixed and paraffin-embedded (FFPE) and the other was conserved in Trizol at −70 °C. PB samples from 27 healthy donors without any known systemic or liver disease and/or HIV, and with normal biological liver test as well as absence of anti-HCV antibodies, were also included as uninfected controls for Flow cytometry analysis.

CHC infection diagnosis was based on the presence of anti-HCV antibodies in serum samples and HCV RNA in plasma in at least 2 separate occasions. Patients had no other causes of liver disease, autoimmune or metabolic disorders, hepatocellular carcinoma or co-infection with HBV and/or HIV. Cases with alcohol consumption (men > 30 g/day; women > 20 g/day) were excluded^34^.

Informed consent was obtained from each patient and the study protocol conforms to the ethical guidelines of the 1964 Declaration of Helsinki and its later amendments, as reflected in a priori approval by the Ethics in Research Committees of Ricardo Gutierrez Children Hospital, Hospital Italiano de Buenos Aires, Hospital JM. Ramos Mejía as well as of Hospital General de Agudos “Carlos G. Durand”.

### Histological Analysis

Histological sections were blindly evaluated by two independent pathologists. Inflammatory activity and fibrosis were assessed using the modified Knodell scoring system (Histological Activity Index, HAI) and METAVIR^35^. Each biopsy was categorized according to HAI, as minimal (≤ 3), mild (4–6), moderate (7–12) or severe hepatitis (>12), and according to METAVIR as significant fibrosis (≥2) or advanced fibrosis (≥3).

Since METAVIR is specially designed for HCV biopsy analysis, it is more accurate to determine fibrosis stages, while modified Knodell classifies inflammatory activity and hepatitis with a deeper characterization of damage localization and severity. We selected each score to quantify the corresponding parameter.

### Immunohistochemical and fluorescence analysis

Infiltrate characterization was performed using appropriate antibodies: mouse anti-CD20 (L26, ready to use, VENTANA, Roche), rabbit anti-CD4 (SP35, ready to use, VENTANA, Roche), rabbit anti-CD8 (SP57, ready to use, VENTANA, Roche), mouse anti-Foxp3 (236 A/E7, 10 μg/ml, Abcam), mouse anti-Tbet (4B10, 1:250, BD Pharmingen), and goat anti-IL-17A (AF-317-NA, 1:250, R&D Systems). Permeabilization and epitope retrieval were performed using sodium citrate buffer (0.01 M, pH 6) in autoclave during 5 minutes (20 psi). Then, sections were incubated 1 h at 25 °C with each primary antibody except for anti-IL-17A which was incubated 18 h at 4 °C. The staining was performed by applying PolyTek HRP anti-Mouse Polymerized Imaging System (PIR080, ScyTek Laboratories) or ultraView^TM^ Universal DAB (cat. 760-500, VENTANA, Roche) or Cell & Tissue Staining Goat Kit (cat. CTS008, R&D Systems) according to the manufacturer’s instructions. Each staining kit provides the specific blocking buffer; moreover, PBS (Na_2_KPO_4_ 10 mM, KH_2_PO_4_ 1,8 mM, NaCl 137 mM, KCl 2,6 mM, pH 7,4) was used as wash buffer. Tonsil sections were used as positive controls. Isotype controls were also performed on tonsil sections [mouse IgG1 k (B11/6, ab91353, Abcam), mouse IgG2a k (B12/8, ab91361, Abcam), purified rabbit IgG (02–6102, Invitrogen) and polyclonal normal serum goat IgG (AB-108-C, R&D Systems)]. Immunostained and total P-P/I lymphocytes were counted in all portal tracts of the tissue section (400×), and frequencies were calculated as positive/total lymphocytes of the whole specimen. Immunostained lymphocytes were also count in 10 random fields from intralobular areas (400×).

Double CD4/Foxp3 and CD4/IL-17A immunostaining were performed. The first staining procedure started with a retrieval step followed by the slides incubation for 1 h at 25 °C with mouse anti-CD4 (1F6, ready to use, Novocastra ^TM^), then detection was performed with Vectastain Elite ABC and DAB Substrate Kit for Peroxidase (Vector Laboratories Inc). Before Foxp3 immunostaining, a blocking step was done using PBS-BSA 1%, then sections were incubated 1 h at 25 °C with anti-Foxp3 antibody. Detection was performed using an anti-mouse FITC- conjugated antibody (115-095-003, 1:100, Jackson ImmunoResearch) for 18 h at 4 °C. Finally, samples were counterstained and mounted with DAPI/antifade Solution (S7113, Chemicon). PBS was used as the wash buffer. CD4 images were converted to false red colour with the Image-Pro Plus software version 6.0.0.260 and merged with fluorescent ones. Isotype control (mouse IgG1 k, B11/6, ab91353, Abcam) was also performed. In the case of CD4/IL-17A labelling, since CD4 shows a membrane immunostaining and IL-17A a cytoplasmic one, a laser confocal microscope was employed for fluorescence studies to ensure co-localization. After retrieval and blocking step (PBS-BSA 1%, 25 minutes at 25 °C) slides were incubated with rabbit anti-CD4 (SP35, ready to use, VENTANA, Roche) for 30 minutes at 37 °C, and then overnight at 4 °C with anti-IL-17A antibody. The detection was done by a donkey anti-Goat IgG-Alexa Fluor 488 (A-11055, 1: 1000, Invitrogen) for 2 h at 25 °C followed by the goat anti-rabbit Alexa-Fluor 568 (A-11011, 1: 400, Invitrogen) for 2 h at 25 °C. Samples were counterstained and mounted with DAPI/antifade Solution. The images were taken and analyzed by Olympus FV-1000 confocal microscopy and Fiji ImageJ program.

### Quantitative Real-Time Reverse-Transcriptase PCR (qRT-PCR) analysis

Total RNA was isolated from liver samples, using Epicentre Master Pure RNA Purification kit (Illumina), according to manufacturer’s instructions. A DNAse (RQ1 RNAse-free DNAse, Promega) treatment was performed in all RNA samples. cDNA was reversed transcribed from 2 μg of RNA, using random 6-mer oligonucleotides (5 ng/μL) and Superscript II RT kit (Invitrogen, California, USA).

The design and validation of IL-10, IL-17A, TGF-β, IL-6, IFN-γ and TNF-α specific primers are described in Supplementary Table S1. A 1/10 aliquot of the cDNA reaction product was used in duplicate qPCR reactions, and all measurements were averaged. qPCR was performed in a final volume of 25 µl of Fast Start Universal SybrGreen Master Mix (Roche) including 5 µl diluted cDNA using a StepOne real-time (Applied Biosystems). The endogenous HPRT or β-actin genes were used as endogenous controls for sample normalization (reference gene) according to the expression level of the studied gene.

Thermocycler conditions for the IL-6/IL-17A/TNF-α genes included 95 °C for 10 min, followed by 40 cycles at 95 °C for 15 sec and 60 °C for 60 sec. For the IFN-γ/TGF-β/IL-10 genes, the annealing temperature changed to 58 °C. In order to verify the specificity of the PCR products, melting curve analysis was performed from 60 °C to 95 °C with 0.3 °C/sec intervals and stepwise fluorescence acquisition. The efficiency of each qRT-PCR reaction ranged between 0.9 and 1.1. The normalized transcription values were calculated by the Pfaffl Method^36^. Results were expressed as fold change (FC).

### Cell isolation and flow cytometric analysis

B lymphocytes (CD19^+^), CTL (CD3^+^CD8^+^) and Th lymphocytes (CD3^+^CD4^+^) frequencies were assessed using anti-CD19-APC-Cy7 (cat. 557791, BD Biosciences), anti-CD3-APC (cat. 561810, BD Biosciences), anti-CD8-APCH7 (cat. 560273, BD Biosciences), anti-CD4 PerCP-Cy5.5 (cat. 341654, BD Biosciences) and anti-CD45-V500 (cat. 560777, BD Biosciences) on fresh heparinized blood. Then PBMCs were isolated from the remnant aliquot of fresh heparinized blood by Ficoll-Paque (Amersham Bioscience) to assess Th subpopulations. Treg (CD4^+^/CD25^hi^/Foxp3^+^) lymphocytes were evaluated using Foxp3 staining kit (cat. 560047, BD Pharmingen; isotype control: cat. 557702, BD Pharmingen), while Th1 (CD4^+^/ IFN-γ^+^) and Th17 (CD4^+^/IL-17A^+^) using Human Th1/Th2/Th17 Phenotyping Kit (cat. 560751, BD Pharmingen), according to manufacturer’s instructions. The last was performed both on basal PBMC and on anti-CD3 (0.166 ng/µl), IL-2 (0.08 pg/µl) stimulated PBMC for 18hs. Gating strategies are shown in Supplementary Fig. 7.

Data were collected on a BD FACSCantoTM II cytometer (BD Bioscience) and analysed using BD FACSDiva™ Software.

### Statistical analysis

Statistical analysis was performed using GraphPad Prism version 5.01 (GraphPad Software Inc). To compare the means between groups, ANOVA or Student’s t test were performed. To determine differences between groups not normally distributed, medians were compared using the Mann-Whitney U test or Kruskal Wallis test. Pearson’s correlation coefficient was used to measure the degree of association between continuous, normally distributed variables. The degree of association between non-normally distributed variables was assessed using Spearman’s nonparametric correlation. P values <0.05 were considered statistically significant.

## Electronic supplementary material

Supplementary material
