# Supplementary material for: Chronic hepatitis C liver microenvironment: role of the Th17/Treg interplay related to fibrogenesis
Source: Sci Rep. 2017 Oct 16;7:13283. doi: 10.1038/s41598-017-13777-3 (PMC5643436; doi:10.1038/s41598-017-13777-3)
Supplement: Supplementary file 1 — Supplementary material [file 41598_2017_13777_MOESM1_ESM.pdf]

Chronic hepatitis C liver microenvironment: role of the Th17/Treg interplay related to fibrogenesis

Daniela Alejandra Rios[a*¥], Pamela Valva[a¥], Paola Cecilia Casciato[b], Silvia Frias[c], María Soledad Caldirola[d], María Isabel Gaillard[d], Liliana Bezrodnik[d], Juan Bandi[b], Omar Galdame[b], Beatriz Ameigeiras[c], Diana Krasniansky[e], Carlos Brodersen[e], Eduardo Mullen[f], Elena Noemí De Matteo[a], María Victoria Preciado[a].

[¥] Equally contributed

[a] Instituto Multidisciplinario de Investigaciones en Patologías Pediátricas (IMIPP- CONICET-GCBA) Laboratorio de Biología Molecular, División Patología, Hospital de Niños Ricardo Gutiérrez, Gallo 1330, C1425EFD, Buenos Aires, Argentina.

[b] Unidad de Hepatología, Hospital Italiano de Buenos Aires; Juan D Perón 4190, C1181ACH, Buenos Aires, Argentina.

[c] Unidad de Hepatología, Hospital Ramos Mejía; Urquiza 609, CP1221, Buenos Aires, Argentina.

[d] Instituto Multidisciplinario de Investigaciones en Patologías Pediátricas (IMIPP- CONICET-GCBA), Departamento de Inmunología, Hospital de Niños Ricardo Gutiérrez; Gallo 1330 C1425EFD, Buenos Aires, Argentina.

[e] Unidad de Hepatología, Hospital General de Agudos "Carlos G. Durand"; Av Díaz Vélez 5044, C1405DCS, Buenos Aires, Argentina

[f] División Patología, Hospital Italiano de Buenos Aires; Juan D Perón 4190, C1181ACH, Buenos Aires, Argentina.

[*] **Corresponding author:** Daniela Alejandra Ríos; Instituto Multidisciplinario de Investigaciones en Patologías Pediátricas (IMIPP- CONICET-GCBA) Laboratorio de Biología Molecular, División Patología, Hospital de Niños Ricardo Gutiérrez, Gallo 1330, C1425EFD, Buenos Aires, Argentina. Fax: +54-11-4962-9138. E-mail: rios.daniela.89@gmail.com

Supplementary table S1: Primer sequences used in SybrGreen qRT-PCR

Target	Primer Sequences (5’-3’)		Product Length (nt)
**IL-10**	F	GCTGTCATCGATTTCTTCCC	111
	R	ACAAAGCCATGAGTGAGTTTGA	
**IL-17A**	F	AACGATGACTCCTGGGAAGAC	99
	R	CCTGGATTTCGTGGGATTGTG	
**TGF-β**	F	CTTCCAGCCGAGGTCCTT	92
	R	CCCTGGACACCAACTATTGC	
**IL-6**	F	AGTGAGGAACAAGCCAGAGC	99
	R	GTCAGGGGTGGTTATTGCAT	
**IFN-γ**	F	GAGTGTGGAGACCATCAAGGA	127
	R	GTATTGCTTTGCGTTGGACA	
**TNF-α**	F	CTGCTGCACTTTGGAGTGAT	93
	R	AGATGATCTGACTGCCTGGG	
**HPRT**	F	ATGGGAGGCCATCACATTGT	77
	R	ATGTAATCCAGCAGGTCAGCAA	
**β-actin**	F	CCACACTGTGCCCATCTACG	131
	R	CCGTGGTGGTGAAGCTGTAG	

F: forward primer; R: reverse primer

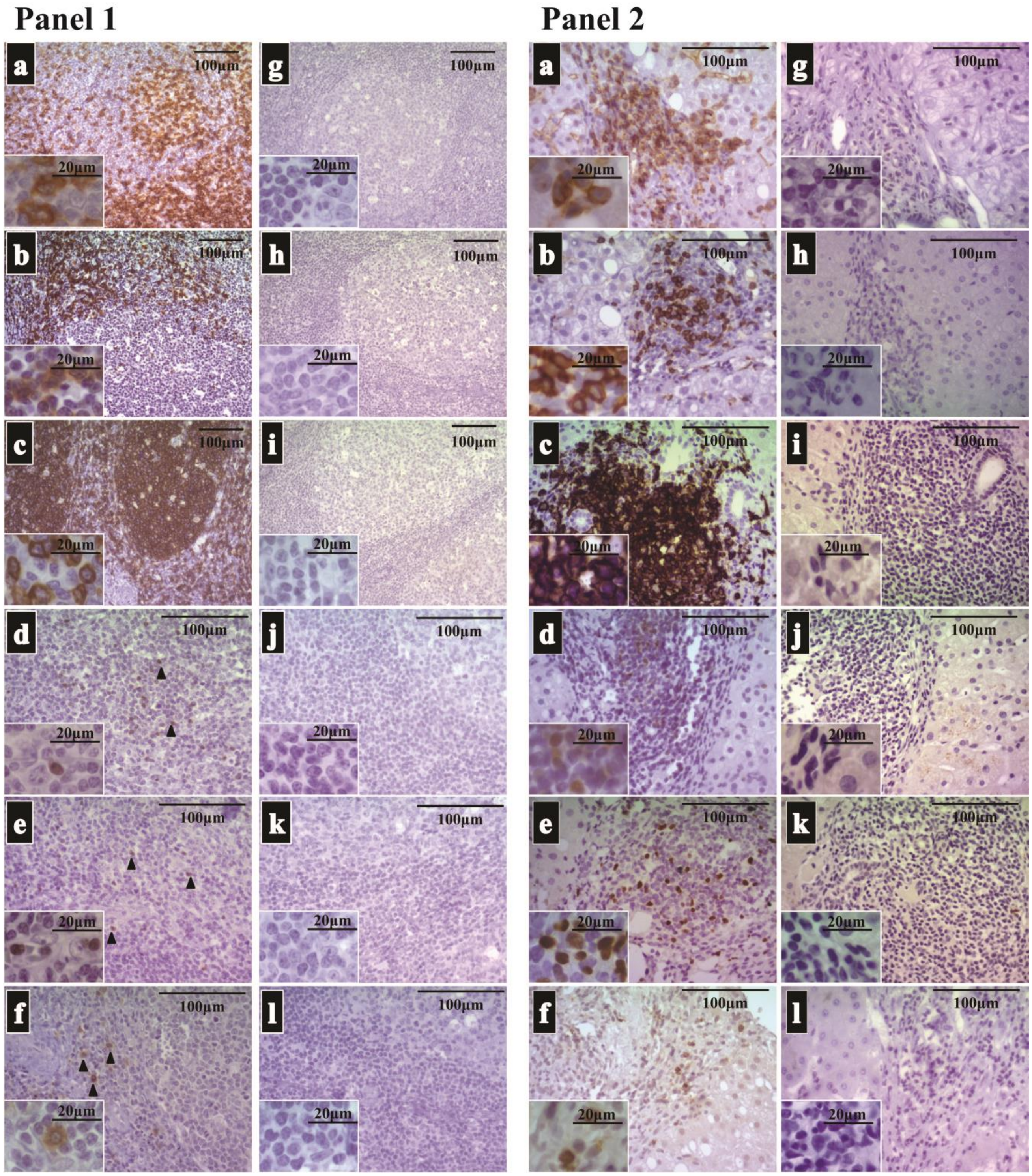

Supplementary Fig. 1 CD4$^+$, CD8$^+$, CD20$^+$, Tbet$^+$, Foxp3$^+$, IL-17A$^+$ lymphocytes immunostaining. Panel 1 shows positive and isotype controls on tonsil sections. Panel 2 shows positive and isotype controls on liver sections. a) CD4$^+$, b) CD8$^+$, c) CD20$^+$, d) Tbet$^+$, e) Foxp3$^+$, and f) IL-17A$^+$ lymphocytes. g), h), i), j), k) and l) show the isotype controls for a), b), c), d), e) and f), respectively.

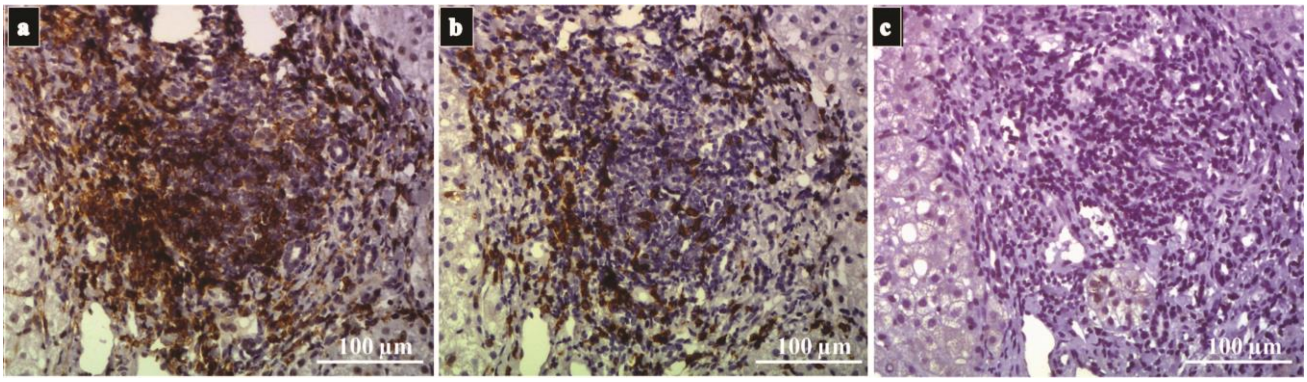

Supplementary Fig. 2 $CD4^+$ and $CD8^+$ lymphocytes distribution in P-P/I areas. A portal infiltrate showing a) $CD4^+$ lymphocytes in the centre and interface area b) $CD8^+$ lymphocytes with peripheral localization within the lymphoid aggregate and c) isotype control.

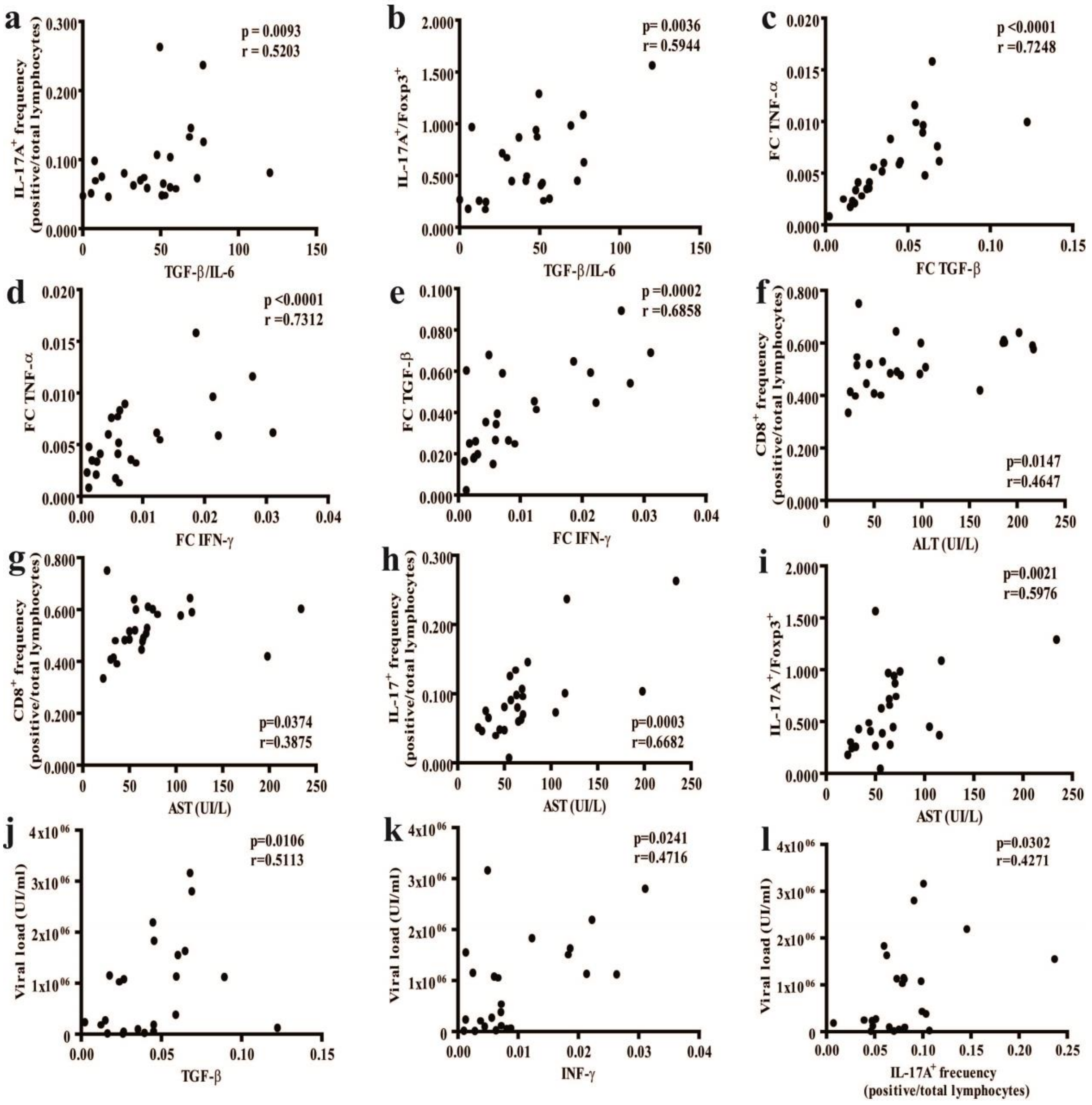

Supplementary Fig. 3 Correlation among cytokine expression levels, intrahepatic lymphocyte frequency, clinical, and virological parameters of CHC patients. a) Correlation between IL-17A$^+$ lymphocyte frequency and TGF-β/IL-6 ratio; b) Correlation between IL-17A$^+$/Foxp3$^+$ lymphocytes ratio and TGF-β/IL-6 ratio, c-e) correlation among TNF-α, IFN-γ and TGF-β expression level; f- i) correlation between transaminase level and CD8$^+$ lymphocyte frequency, IL-17A$^+$ lymphocyte frequency and IL-17A$^+$/Foxp3$^+$ lymphocytes ratio, respectively; j-l) correlation between viral load and TGF-β, IFN-γ, and IL-17A$^+$ lymphocyte frequency, respectively. Frequencies were calculated as immunostained P-P/I lymphocytes/ total P-P/I lymphocytes in all portal tracts of the tissue section (400×). FC: fold change. Spearman's nonparametric correlation (a, d-l) and Pearson's correlation coefficient (b and c) were used to measure the degree of association between the studied parameters.

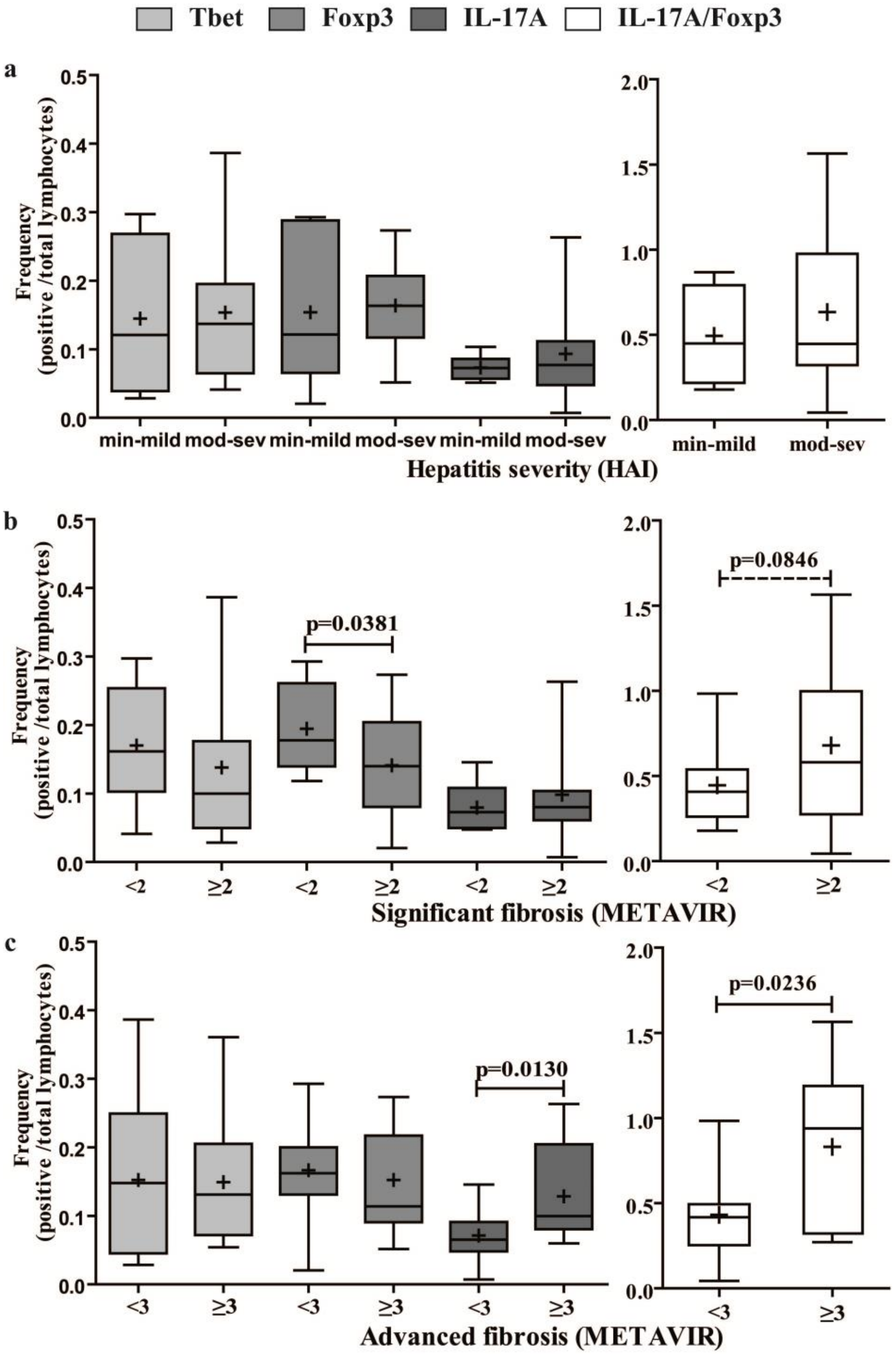

Supplementary Fig. 4 Intrahepatic Th subsets frequency related to histological liver damage parameters. $Tbet^+$, $Foxp3^+$, IL-$17A^+$ lymphocyte frequency as well as IL-$17A^+$/$Foxp3^+$ratio related to a) hepatitis severity, b) significant and c) advanced fibrosis. Hepatitis severity (min: minimal; mod: moderate, sev: severe) according to HAI. Significant (F ≥2) and advanced (F ≥3) fibrosis according to METAVIR. The results are depicted in box plots. Horizontal lines within boxes indicate medians. Horizontal lines outside the boxes represent the 5 and 95 percentiles. Mean is indicated as +. Frequencies were calculated as immunostained P-P/I lymphocytes/ total P-P/I lymphocytes in all portal tracts of the tissue section (400×). The Mann-Whitney U-test and unpaired t-test were used to compare sets of data..

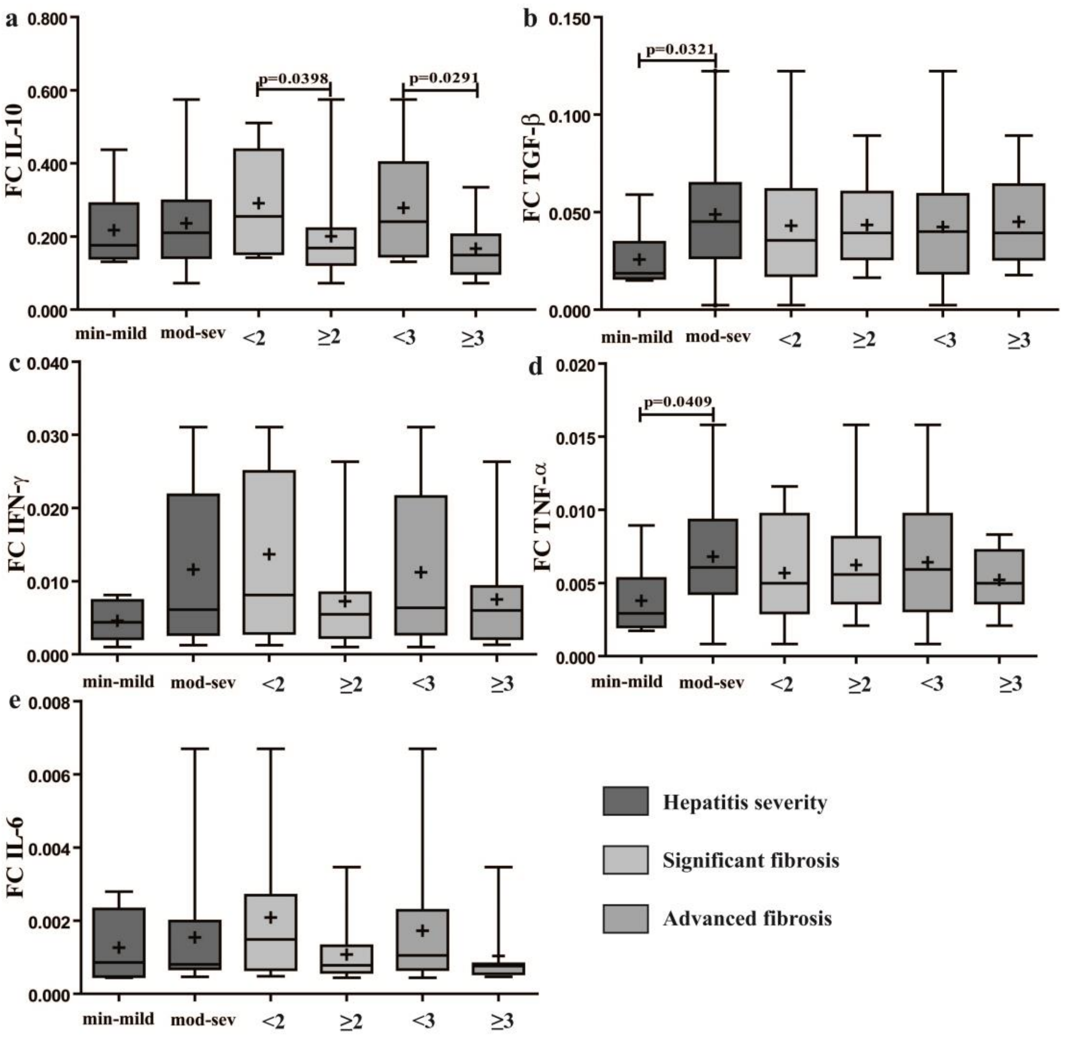

Supplementary Fig. 5 Cytokine expression levels in the liver milieu of CHC patients related to liver damage. a) IL-10, b) TGF-β, c) IFN-γ, d) TNF-α, and e) IL-6 expression levels related to hepatitis severity, significant and advanced fibrosis. Hepatitis severity (min: minimal; mod: moderate, sev: severe) according to HAI. Significant (F ≥2) and advanced (F ≥3) fibrosis according to METAVIR. The results are depicted in box plots. Horizontal lines within boxes indicate medians. Horizontal lines outside the boxes represent the 5 and 95 percentiles. Mean is indicated as +. FC: fold change. The Mann-Whitney U-test and unpaired t-test were used to compare sets of data.

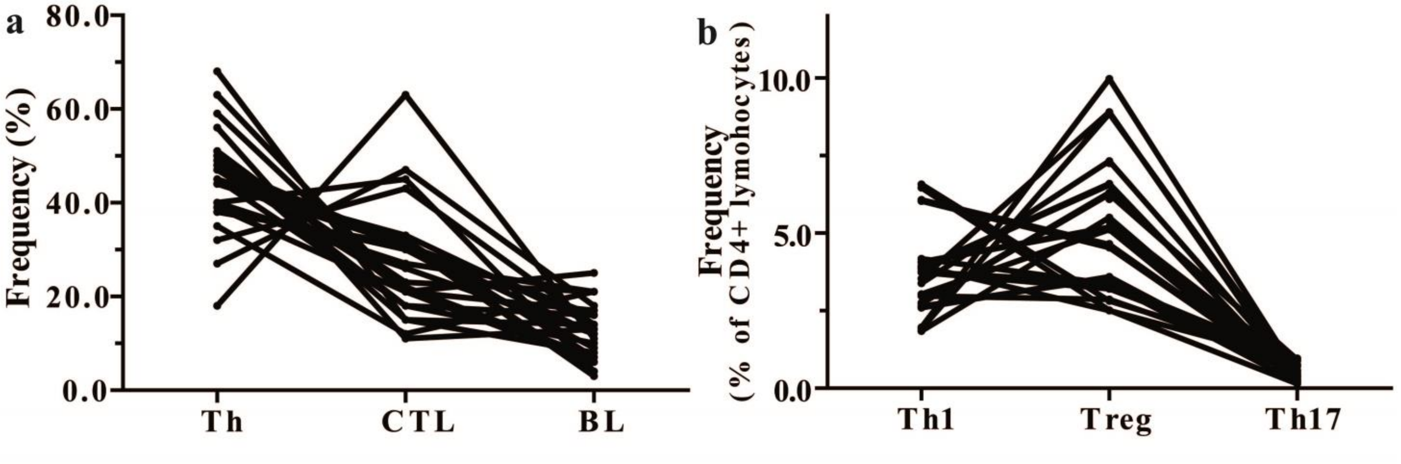

Supplementary Fig. 6 Peripheral lymphocyte populations in CHC patients. a)Th, CTLs and B lymphocytes frequency for each patient, b) Th1, Treg and Th17 lymphocytes frequency for each patient.

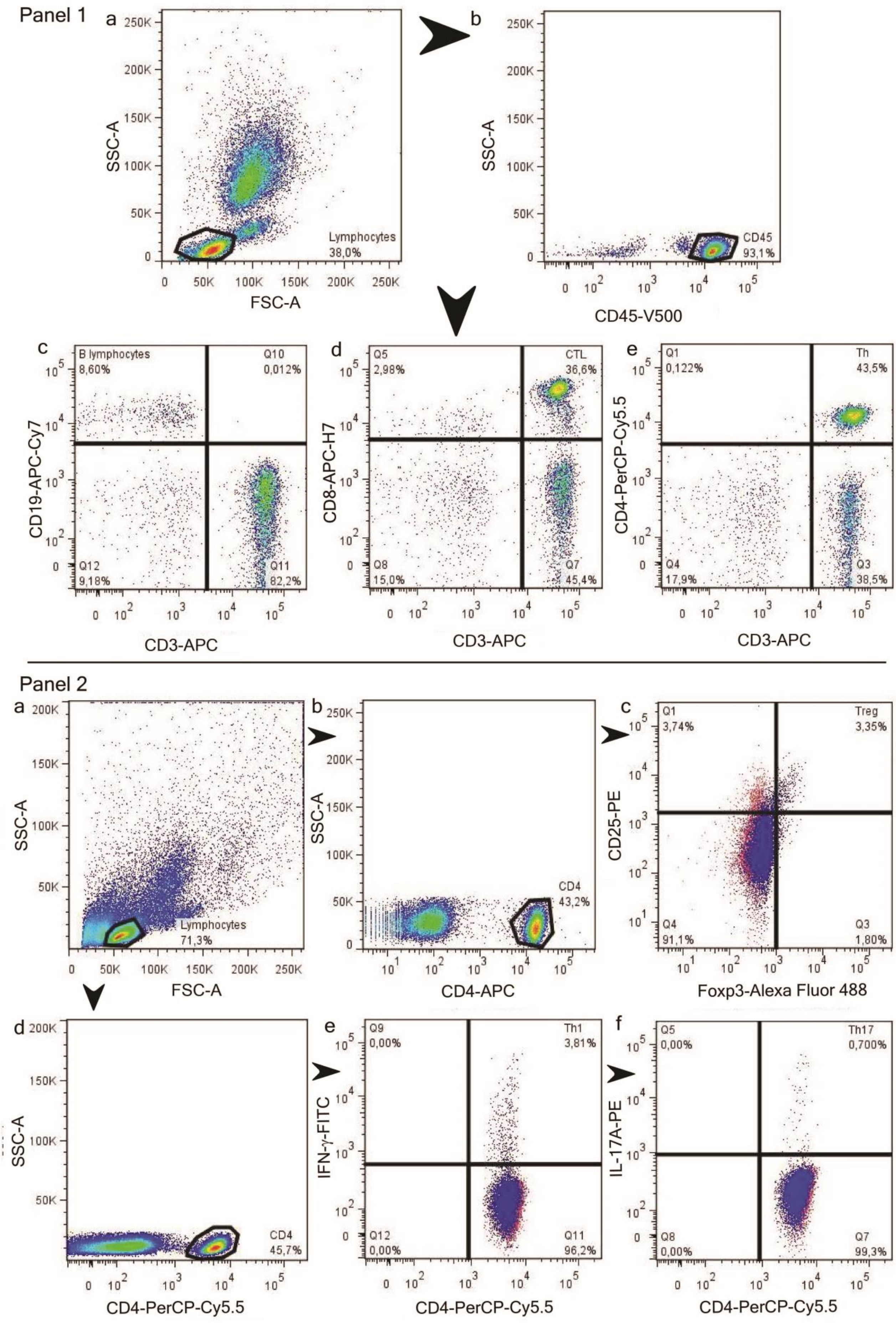
Panel 1
a
SSC-A
FSC-A
Lymphocytes
38,0%
b
SSC-A
CD45-V500
CD45
93,1%
c
CD19-APC-Cy7
CD3-APC
B lymphocytes
8,60%
Q10
0,012%
Q12
9,18%
Q11
82,2%
d
CD8-APC-H7
CD3-APC
Q5
2,98%
CTL
36,6%
Q8
15,0%
Q7
45,4%
e
CD4-PerCP-Cy5.5
CD3-APC
Q1
0,122%
Th
43,5%
Q4
17,9%
Q3
38,5%
Panel 2
a
SSC-A
FSC-A
Lymphocytes
71,3%
b
SSC-A
CD4-APC
CD4
43,2%
c
CD25-PE
Foxp3-Alexa Fluor 488
Q1
3,74%
Treg
3,35%
Q4
91,1%
Q3
1,80%
d
SSC-A
CD4-PerCP-Cy5.5
CD4
45,7%
e
IFN-γ-FITC
CD4-PerCP-Cy5.5
Q9
0,00%
Th1
3,81%
Q12
0,00%
Q11
96,2%
f
IL-17A-PE
CD4-PerCP-Cy5.5
Q5
0,00%
Th17
0,700%
Q8
0,00%
Q7
99,3%

Supplementary Fig. 7 Gating strategies for flow cytometry analysis. Panel 1: B lymphocytes, CTLs and Th frequency on fresh heparinized blood. Lymphocyte selection by a) SSC-A and FSC-A, and b) CD45 expression. Lymphocyte characterization by c) CD3-CD19$^+$ (B lymphocytes), d) CD3$^+$CD8$^+$ (CTL) and e) CD3$^+$CD4$^+$ (Th cells). The percentage of each population is indicated. Panel 2: Th subpopulations on PBMCs sample. a) lymphocyte selection by SSC-A and FSC-A, b) CD4$^+$ lymphocytes selection for Treg characterization, c) CD4$^+$CD25hiFoxp3$^+$ (Treg) cells identification (blue) (isotype control staining is also shown in red), d) CD4$^+$ lymphocytes selection for Th1 and Th17 characterization; e) CD4$^+$/ IFN-γ^+ (Th1) lymphocytes and f) CD4$^+$/IL-17A$^+$ (Th17) lymphocytes identification after anti-CD3/IL-2 stimulation (blue) (unstimulated PBMCs are also shown in red). The percentage of each population is indicated.
